# Predictors of Abortion Attitudes in Medical Students After the Reversal of Roe v. Wade

**DOI:** 10.7759/cureus.35421

**Published:** 2023-02-24

**Authors:** Robin J Jacobs, Michael N Kane, Kristina Fritz

**Affiliations:** 1 Medical and Behavioral Research; Health Informatics; Medical Education, Nova Southeastern University, Fort Lauderdale, USA; 2 Social Work, Florida Atlantic University, Boca Raton, USA; 3 Dr. Kiran C. Patel College of Osteopathic Medicine, Nova Southeastern University, Fort Lauderdale, USA

**Keywords:** perceptions, attitudes, religion, medical student, medical education, pro-life, pro-choice, knowledge, religiosity, abortion

## Abstract

Background

On June 24, 2022, the United States (U.S.) Supreme Court ruled in *Dobbs v. Jackson Women’s Health *that the Constitution does not grant women the right to abortion. This new ruling may have a profound impact on not only the attitudes of medical trainees but the nature in which they are trained when it comes to abortion practices, indications, or procedures. Some clinics where medical schools provide first-hand abortion experience have closed. As a surge of extreme restrictions on abortion has been seen in certain states in the U.S., medical schools and residency programs need to address these issues to ensure future physicians are adequately prepared. The purpose of this study was to assess factors that influence medical students’ attitudes toward abortion, specifically knowledge about abortion, religiosity, and philosophical group affiliation regarding abortion (i.e., “pro-choice vs. “pro-life”).

Methodology

This cross-sectional study collected data from a convenience sample of 413 medical students between October and December 2022 via an online, anonymous questionnaire. Major study variables as depicted in the published literature led to the development of the Abortion Attitudes Questionnaire (AAQ) for medical students. The AAQ contained validated scales to assess the contribution of levels of abortion knowledge and religiosity as well as sample characteristics on attitudes about abortion in medical students. Speakman rank correlation and linear multivariate regression were used for hypothesis testing to explore the contributions of the dependent variables to attitudes about abortion in medical students.

Results

The mean age of the participants was 25.8 years (SD = 2.96; range = 21-45 years). Linear regression modeling indicated that religiosity, abortion knowledge, being a woman, and group affiliation regarding abortion (“pro-choice” or “pro-life”) explained a significant amount of the variance (60%) in abortion attitudes scores in medical students. A significant regression equation was found, F(6,373) = 83.570, p < 0.0001, R^2 ^= 0.603, R^2^ adjusted = 0.611). Less religiosity, greater abortion knowledge, being a woman, and identifying as “pro-choice” significantly contributed to more positive attitudes toward abortion in this sample of medical students. Interestingly, while moderately correlated with abortion attitudes (r = 0.436,p < 0.01), the single item regarding the importance of religion in one’s life did not contribute to the model.

Conclusions

The present study is the first, to our knowledge, to identify medical student characteristics (i.e., sex, “pro-choice” vs. “pro-life” group affiliation, level of knowledge about abortion, and religiosity) as indicators of abortion attitudes. With the reversal of *Roe v. Wade*, attention must be given to the possible change in medical students’ attitudes toward abortion (as well as any newly developed constraints on clinical training) and to ensure the provision of comprehensive education as U.S. states will determine the limits of these practices and procedures. While further research in this area is needed, findings from this study can help assess students’ attitudes about abortion and guide medical education efforts to better prepare tomorrow’s obstetrics and gynecology physicians.

## Introduction

On June 24, 2022, the United States (U.S.) Supreme Court ruled in *Dobbs v. Jackson Women’s Health* that the Constitution does not grant women the right to abortion [1]. This new ruling may have a profound impact on not only the attitudes of medical trainees but the nature in which they are trained when it comes to abortion practices, indications, or procedures. Some clinics where medical schools provide first-hand abortion experience have closed. As a surge of extreme restrictions on abortion has been seen in certain states in the U.S., medical schools and residency programs need to address these issues to ensure future physicians are adequately prepared.

Abortion in the United States

More common than an appendectomy, prior to the supreme court ruling, abortion was one of the most common medical procedures in the U.S. [2,3]. Approximately 30% of women will undergo an abortion before their mid-forties, and nearly half will seek an abortion in their lifetimes [3]. The Centers for Disease Control and Prevention (CDC) defines abortion as “an intervention performed within the limits of state law by a licensed clinician (e.g., a physician, nurse-midwife, nurse practitioner, or physician assistant) intended to terminate a suspected or known intrauterine pregnancy and that does not result in a live birth” [4]. The two types of induced abortion services deemed safe in the U.S. are procedural and medical [5]. Medical abortion utilizes prescription medications, such as mifepristone and misoprostol, to block progesterone and empty the uterus during the first eight weeks of pregnancy [5,6] whereas procedural abortions require surgically entering the uterus to remove fetal tissue. In the U.S., the majority of abortions are procedural and completed at or before 13 weeks gestation. However, medical abortions have been increasing since the Food and Drug Administration’s (FDA) approval of mifepristone in 2000 and are likely to continue to increase after the *Dobbs v. Jackson Women’s Health* court ruling [5,6]. A recent study found that after the Supreme Court’s draft decision leaked and after the decision came down, there were distinct increases in medication provided to safely end a pregnancy at home [7]. This trend in medical abortion is conjectured to continue as restrictions on procedural abortions increase because, regardless of changing abortion laws, women still need and seek abortion care [7].

In 2020, a total of 620,327 abortions were reported to the CDC; the abortion rate was 11.2 abortions per 1,000 women, and the abortion ratio was 198 abortions per 1,000 live births [4]. Since 2011, there has been an overall decrease in the total number, rate, and ratio of reported abortions [2,4]. A few studies suggest that the decrease in abortions could be due to decreased incidence of unwanted pregnancy, as nearly half of all unintended pregnancies end in abortion [2,3]. The decrease in unintended pregnancy is thought to be due to the increasing use of long-acting reversible contraceptive (LARC) methods, an increase in female sterilization, an increase in correct condom use among sexually active adults in the U.S., and an increase in *self-managed* abortions [2]. A self-managed or self-induced abortion describes the ending of a pregnancy without healthcare professional supervision [2]. Currently, medical/medication abortion via mifepristone is not considered self-managed because the FDA limits its distribution to registered providers in clinics, hospitals, and medical offices, and it cannot be dispensed at pharmacies [6]. Self-managed abortions have historically and continue to be unsafe because of physically inflicted damage to the uterus and other body tissues or unsupervised medical abortion, preciously labeled “back alley” or “coat hanger” abortions [2,6]. Despite the decreases in the number of abortions legally provided, procedural and medical abortion training and practice are necessary to provide adequate care to the patient for both their health and well-being [3].

Despite criticisms, abortions are safe and have been shown to help women. The National Academies of Sciences, Engineering, and Medicine found that all legal methods of abortion are safe and effective with rare complications [3]. Abortion procedures have fewer complications than routine procedures such as colonoscopies, wisdom tooth removal, and tonsillectomies and do not typically have lasting consequences on the mental or physical health of women [3,8]. In fact, researchers have found that women who are denied an abortion are more likely to suffer physical and mental harm [8]. Studies that compared women who were able to have an abortion to those who wanted an abortion but were unable to attain one found that women who did not have an abortion were more likely to suffer from depression, anxiety, and poorer physical health five years later [9-11].

Studies have also shown that locations that limit access to abortion do not decrease the number of unintended pregnancies, decrease the demand for abortion, or improve women’s health, and are more likely to have fewer safe abortions with more complications [9]. Higher maternal mortality rates are also seen in places that limit abortion, as the risk of death from childbirth is 14 times greater than that from a legal abortion [2]. The U.S. has the highest mortality rate of any industrialized country, nearly one death per 1,000 cases of childbirth, and that mortality rate is even higher in states that are more restrictive on abortion [9,12]. It should also be noted that lower income, younger, and racial and ethnic minority women experience unintended pregnancy at significantly greater rates than their non-Hispanic white counterparts and are also disproportionately affected by abortion policy, with Black women having the highest maternal mortality ratio [9,12].

Although there are ample health benefits to providing legal abortions, there have been increasing restrictions and bans placed on abortion since the *Roe v. Wade* decision in 1973 and have continued after the *Dobbs v. Jackson Health decision* in 2022 [2,9]. The current rhetoric in the media with regard to abortion as a procedure with medical consequences to women and the fetus, including depression and infertility, was first used by the primarily if not all-male American Medical Association (AMA) as a response to women’s rights movements to maintain power in and control of medical practice, suppress competition of (mainly female) midwives, and as a reaction to women lobbying for rights to attend medical school and study obstetrics and gynecology (OBGyn) [3]. More recent studies have shown that while abortion oppositionists rally behind that reducing harm argument (to the woman and fetus), conservative values of purity, gender norms, and morality, as well as the maintenance of spiritual perfection, is a more accurate predictor of abortion opposition [13]. Interestingly, conservative moral values and the protection of *unborn life* that are typically associated with anti-abortion sentiment were not shared by religious organizations until 1979 [3]. Of note, in 1974, after the *Roe v. Wade* decision, the Southern Baptist Convention issued a statement that abortion should be available for rape; fetal deformity; emotional, physical, or mental well-being threats to the mother; and incest, and also encouraged their congregations to support legislation that protected abortion [3].

Physicians and abortion

Despite historical political motives, abortion support continues to be aligned with personal moral values, and physicians are no exception. Although 61% of Americans believe that abortion should be legal in all/most cases, according to Pew Research Center, women’s ability to get an abortion is determined by their geographic location, income/wealth, and their physician’s opinion [3,14]. With increasing state abortion restrictions, the number of facilities that offer abortions is declining, with some states only having one functioning abortion clinic [5]. This effect is more pronounced as approximately 58% of women live in states with more restrictions on abortion, such as those in the southern regions [5]. In almost every state, laws have been enacted that allow healthcare professionals and institutions to refuse abortions to patients [3]. A study that surveyed 1,800 practicing OBGyn physicians found that out of the 97% who were asked to provide abortions, only 14% performed them, with the majority performed by young physicians, those located in the Northeast or West, in highly urban zip codes, or those who identified as Jewish [13].

Other surveys found that OBGyn physicians were less likely to support abortion than physicians of other specialties [14,15]. Another study comparing Maternal-Fetal Medicine (MFM) physicians, a subspecialty of OBGyn, and Family Care Practitioners (FCPs) found that MFMs were more likely to support abortion than FCPs, which is noteworthy as FCPs provide a large amount of family planning care and have the ability to prescribe medical abortions and perform abortions [3,16]. Overall, regardless of specialty, the religion of the provider greatly determined whether or not the physician provided abortion services [3,16]. Studies have found that 45-68% of physicians agreed that religious/spiritual beliefs played a role in their medical practice and affected options offered to their patients [16]. Support for abortion also changed with the situation, with both religious and non-religious physicians more likely to support abortion if fetal abnormalities or health risks were present, whereas religious physicians found social issues, such as economics, undesired pregnancy, or current family relationships, as an inappropriate reason for abortion [3]. The physician’s religion and religiosity also contributed to their willingness to provide abortions, with Catholics, Evangelical Protestants, non-Evangelical Protestants, and physicians with high religious motivation least likely to provide abortions [17]. To address the low number of practitioners providing abortion, an investigation into medical school curricula and future physicians’ opinions on abortion are important to help predict future medicine and install interventions to help address the medical need of the U.S. population.

Abortion knowledge among medical students

Insufficient abortion training may dissuade a provider from performing an abortion when practicing. Studies have found that a large reason why providers would not assist or provide medical abortions is because of no training or insufficient training [3]. Current medical education for future physicians regarding abortion seems to be decreasing over time, and many students want more training. Studies show that when medical students are exposed to abortion in training, they are more likely to provide abortion in their own practice [3,18,19]. Since the 1980s, fewer physicians have been performing abortions, but it is hard to determine how much of that is due to decreases in formal education on the topic [3]. From 1974 to 2008, the percentage of abortions that took place at non-hospital clinics increased from 61% to 95%, resulting in less exposure to and coverage of abortions in medical school with traditional hospital training [3,15]. A study conducted to assess preclinical, third, and fourth years abortion curricula at 78 accredited U.S. medical schools found that 44% had no formal abortion education, 19% had one abortion-specific lecture, 11% included discussions or clinical abortion care experience prior to clinical rotations, and only 45% were offered a clinical abortion experience [3]. Geographic location also seemed to determine exposure to abortion and other women’s health topics. Elective abortions and some contraception were less likely to be addressed in the South compared to other U.S. regions [3].

While medical student knowledge about abortion may be a predictor of whether they will provide abortions when in practice, students’ assessment as to whether they had a sufficient abortion education seemed to vary based on their attitudes toward abortion, with those who were pro-choice and had the intention to provide abortion associated with an increased desire for more training [3,20-22].

Medical students’ attitudes toward abortion

Although medical students are more supportive of abortion than students of other disciplines, such as science and engineering, their opinions on abortions are not homogenous [23,24]. Medical students’ opinion on abortion seems to differ based on the patient’s reasons for undergoing an abortion [3]. Similar to the general U.S. population, there are students who are *true believers* of abortion or against abortion and those whose support is conditional [13]. A true believer is someone who, regardless of the situation, would support or not support abortion, termed pro-choice and pro-life, respectively. However, nearly half of Americans are not *true believers* and will determine whether an abortion is warranted based on the situation, such as in instances of rape or health risks [1].

The largest difference between the pro-choice true believers and everyone else is support for elective abortion, where the woman does not want the child, the mother is in extreme poverty, and the mother is not in a committed relationship with the man [13]. Most individuals in the U.S. support abortion for medical/trauma-based reasons, such as pregnancy would jeopardize the women’s health, fetal defect, and pregnancy as a result of rape [13]. Researchers found that only 28.6-33.1% of medical students believed that abortion is appropriate in all circumstances, and another 38-54% would perform an abortion when sought after for medical reasons rather than non-medical/elective reasons [3]. While this percentage may vary slightly depending on the study, overall, medical students are more likely to support abortion if for medical reasons rather than non-medical ones [3].

The timing of abortion can also affect abortion attitudes. Although the majority of abortions are done prior to 13 weeks, and most questionnaires do not distinguish among dates of abortion, third-trimester abortions are very seldom supported by all groups [3]. As with differing situational opinions regarding abortion, medical students’ attitudes toward abortion differ based on religious affiliation, religiosity, geographic location, age, gender, political alignment, and other personal characteristics.

Religion/religiosity and abortion attitudes

Similar to physicians and the majority of medical students in the U.S. who identify as religious tend to support abortion less [3,5,13]. Religion has been found to be one of the strongest predicting factors of abortion attitudes for medical students [3]. Additionally, the religious denomination that the student identifies with has been found to relate to abortion attitudes [3,5,13]. Studies found that medical students, similar to physicians and the U.S. general population, who identified as Catholic, Protestant, or Evangelical generally opposed abortion and were more likely to be pro-life *true believers*, whereas students who identified as Jewish or non-religious were more likely to support abortion and identify as pro-choice [3,13,18,19,23]. Research regarding the abortion attitudes of individuals and medical students who identify as Muslim has been done outside of the U.S. and showed that Muslims are more likely to support traumatic abortion and oppose elective abortion than other groups [13,24].

Religiosity has been shown to be a better predictor of abortion attitudes than religious denominations for medical students, physicians, and the U.S. population. Religiosity is used to describe how religious the person is regarding religious activities, such as holidays, religious traditions, and attending religious services. Researchers found that regardless of the medical student’s religion, religious service attendance was significantly negatively correlated with abortion attitudes, even more so than their religious denomination [3,23]. One study showed that younger protestants who are not as active in the church have increasingly pro-choice sentiments compared to practicing and older protestants [3]. This relationship between increased religiosity and increased pro-life attitudes is also seen in Latin America, Poland, New Zealand, Northern Ireland, South Africa, Spain, the United Kingdom, and Slovenia, to name a few [13].

Sex/gender and abortion attitudes

Research on gender identification and abortion attitudes has reported mixed results. Many studies find women to be more likely to support abortion; however, studies also find women to be very polarized, resulting in no significant difference between identified gender or just no association at all [3,13,19,23,25-29]. Some reports indicated that men have expressed more support for abortion than women [13]. Instead, gender roles have been a more significant predictor of abortion attitudes, with individuals who identify with traditional gender roles, especially women who do not work outside the home, being more likely to oppose abortion [3,13].

Political alignment and abortion attitudes

Political alignment is often associated with pro-choice and pro-life true believers and has been associated with differences in abortion support [3,13]. Studies found that individuals who identify as conservative and right-wing are less likely to support abortion, while those who identify as liberal and left-wing or left-leaning on the Likert scale are more likely to support abortion rights [13,23]. Additionally, the commitment to partisan values has an effect on abortion support. Individuals who are more ambivalent and have a weaker commitment to their political party ideology are less likely to have strict pro-choice or pro-life views [29].

Although multiple studies have sought to determine factors that influence medical students’ attitudes toward abortion, there is limited data and analysis of medical students in the U.S., and none to date after the 2022 Supreme Court ruling that the Constitution does not protect the abortion rights of women. Within this context, we sought to assess factors that influence medical students’ attitudes toward abortion, specifically knowledge about abortion, religiosity, and philosophical group affiliation regarding abortion (i.e., “pro-choice vs. “pro-life”).

Research question and hypothesis

It is important to document and interpret the attitudes about abortion among future physicians. This study thus sought to answer the question: What factors influence medical students’ attitudes about abortion? Specifically, will knowledge about abortion, religiosity, sex, and certain ideologies about abortion make significant contributions to medical students’ attitudes toward abortion? It was thus hypothesized a priori that higher levels of knowledge about abortion, less religiosity, being a woman, feeling that religion is not important in one’s life, and “pro-choice” group affiliation regarding abortion would make significant contributions to positive attitudes toward abortion in medical students.

## Materials and methods

Overview

After securing approval from the Nova Southeastern University Institutional Review Board, medical students enrolled in a large school of osteopathic medicine in Florida completed an anonymous, online questionnaire that included validated scales to investigate abortion knowledge and religiosity and how these variables might contribute to attitudes toward abortion among medical students. Data were collected from October 12 to December 19, 2022, via (http://projectredcap.org/) a secure, user-friendly web application for building and managing online surveys and databases. Correlation and multiple linear regression analyses were performed using SPSS® version 28 (IBM Corp., Armonk, NY, USA).

Participants and recruitment

Using a cross-sectional, correlational design, a questionnaire was electronically distributed to all medical students in program years one through four using the institution’s student listservs. Of the 1,442 students invited to complete the questionnaire, 488 were returned. Of those, 75 had >33% data missing and were removed, resulting in 413 completed questionnaires.

The self-administered online questionnaire was open from October to December 2022. It took about 10 minutes to complete. Reminder emails were sent to all students after a week and then again after one week, three weeks, and five days prior to the close of the online questionnaire. Anonymity was maintained during the data collection process by using a numerical coding protocol that does not store any identifying information. REDCap was used to collect and store participant data before it was uploaded to SPSS for analysis.

Materials

The Abortion Attitudes Questionnaire

The researchers developed the Abortion Attitudes Questionnaire for medical students, herein referred to as the AAQ. After an extensive review of the literature, major study variables were chosen that were thought to be associated with medical students’ attitudes toward abortion. The 47-item AAQ contained validated measures to assess attitudes toward abortion and religiosity. In addition, abortion knowledge questions and items on participant characteristics were included.

Sample Characteristics

All the participants were osteopathic medical students in years one through four of the Doctor of Osteopathic Medicine program. Thirteen items developed by the researchers were used to assess participants’ race, ethnicity, age, year in medical school, whether they had any personal experience with abortion, and whether they had any training in abortion practices, indications, or procedures. Single items about religion included religious training as a child and current religious affiliation. Participants were also asked if they identified as “pro-life” or “pro-choice” in reference to abortion.

Attitudes Toward Abortion

The 20-item Attitudes About Abortion and Varying Attitude Structures scale assessed abortion attitudes under three domains, namely, availability, moral acceptability, and a woman’s autonomy in an abortion decision. Higher scores indicate more positive attitudes toward abortion, specifically that abortions (1) should be available, (2) are morally acceptable, and (3) a woman should have autonomy in a decision to have an abortion. Conversely, lower scores indicate that abortions (1) should not be available, (2) are morally unacceptable, and (3) a man should have a say in an abortion decision. Responses were scored on a six-category ordinal scale, where 1 = strongly agree, 2 = agree, 3 = somewhat agree, 4 = somewhat disagree, 5 = disagree, and 6 = strongly disagree. Examples of items include “Abortion should be legal,” “Life exists from the moment of conception,” and “Abortion should be entirely the woman’s decision” [30].

Religiosity

Religiosity was measured using the Centrality of Religiosity Scale (CRS-5), a brief five-item measure with scores ranging from 1 to 5, with higher scores indicating a higher level of religiosity [31]. The CRS-5 is a measure of the centrality, importance, or salience of religious meanings in personality. Responses are scored on an ordinal scale, where 1 = very often, 2 = often, 3 = occasionally, 4 = rarely, and 5 = never. Items are categorized by five distinct domains: (1) intellect (“How often do you think about religious issues?”), (2) ideology (“To what extent do you believe that God or something divine exists?”), (3) public practice (“How often do you take part in religious services?”), (4) private practice (“How often do you pray?”), and (5) experience (“How often do you experience situations in which you have the feeling that God or something divine intervenes in your life?”). In addition, a single item was included that asked the participant to rate how important religion was in their life using a four-item Likert response set from very important to not important at all.

Abortion Knowledge

Nine items assessing abortion knowledge were adapted from a measure created by Mullen and colleagues originally tested with psychologists and psychology graduate students using a true/false response set [32]. Examples of knowledge items include “Abortion rates decrease when abortion is illegal,” and “Research demonstrates that adolescents have longer-lasting negative effects from abortion than adult women do.”

Data analysis

All the data were cross-checked for errors (e.g., out-of-range values, missing data, outliers). Surveys with more than 33% of missing data were considered incomplete and thus excluded from the final analysis. Internal consistency (Cronbach’s alpha) for measures was computed for the sample. Internal consistency estimates for measures within the survey were favorable: attitudes toward abortion (α = 0.955), religiosity (α = 0.907), and abortion knowledge (α = 0.650, Kuder-Richardson Formula 20 for dichotomous data). Characteristics of the sample were summarized as frequency and percentage for discrete variables and as means and standard deviation for continuous variables. Summary statistics for the major study variables are reported below. Speakman rank correlation and linear multivariate regression were used for hypothesis testing to explore the contributions of personal characteristics (i.e., sex, group affiliation regarding abortion (“pro-choice” or “pro-life”), abortion knowledge, importance of religion, and religiosity) to attitudes toward abortion in medical students.

## Results

Characteristics of the sample

The characteristics of the sample are presented in Table 1. The mean age of the participants was 25.8 years (SD = 2.96; range = 21-45 years). Regarding medical school program year, 34.1% (n = 41) of the participants were in the first year, 28.6% (n = 118) were in the second year, 22% (n = 91) were in the third year, and 15.3% (n = 63) were in the fourth year.

**Table 1:** Characteristics of the sample (N = 413). *: Valid percent only is reported; missing data were excluded from the calculations unless otherwise noted.

Characteristic	n	Valid percent*
Race		
Asian American Indian or Alaskan Native	86	22.1
Black or African American	10	2.6
White	255	65.4
Other/prefer not to answer	39	10.0
Identify as Hispanic/Latinx	72	18.9
Sex		
Female	251	65.9
Male	130	34.1
Other/Prefer not to answer	32	7.7
Year in the medical school program		
First-year medical student	141	34.1
Second-year medical student	118	28.6
Third-year medical student	91	22.0
Fourth-year medical student	63	15.3
Had personal experience with abortion	59	15.1
Abortion has been a topic of discussion in school training	224	57.4
Received training in abortion practices, indications, or procedure	104	26.7
Group identification regarding abortion		
“Pro-choice”	309	79.2
“Pro-life”	66	19.9
Prefer not to answer	15	3.8
Type of religious tradition received training as a child		
Judaism	26	6.7
Catholicism	139	35.6
Protestant Christianity	56	14.4
Evangelical Christianity	33	8.5
Orthodox Christianity	10	2.6
Jehovah’s Witness	2	0.5
Islam	25	6.4
Buddhism	5	1.3
Hinduism	30	7.7
Atheism/Agnosticism	17	4.4
Spirituality, but no training in any organized denomination or religion	18	4.6
Other	29	7.4
Current religious affiliation		
Jewish	22	5.6
Catholic	62	15.9
Protestant Christian	28	7.2
Evangelical Christian	26	6.7
Orthodox Christian	8	2.1
Jehovah’s Witness	19	4.9
Muslim	6	1.5
Buddhist	15	3.8
Hindu	93	23.8
Spiritual, but not religious	87	22.3
Atheist/Agnostic	24	6.2
Spirituality, but no training in any organized denomination or religion	390	5.6
Other	22	15.9

**Figure 1:**
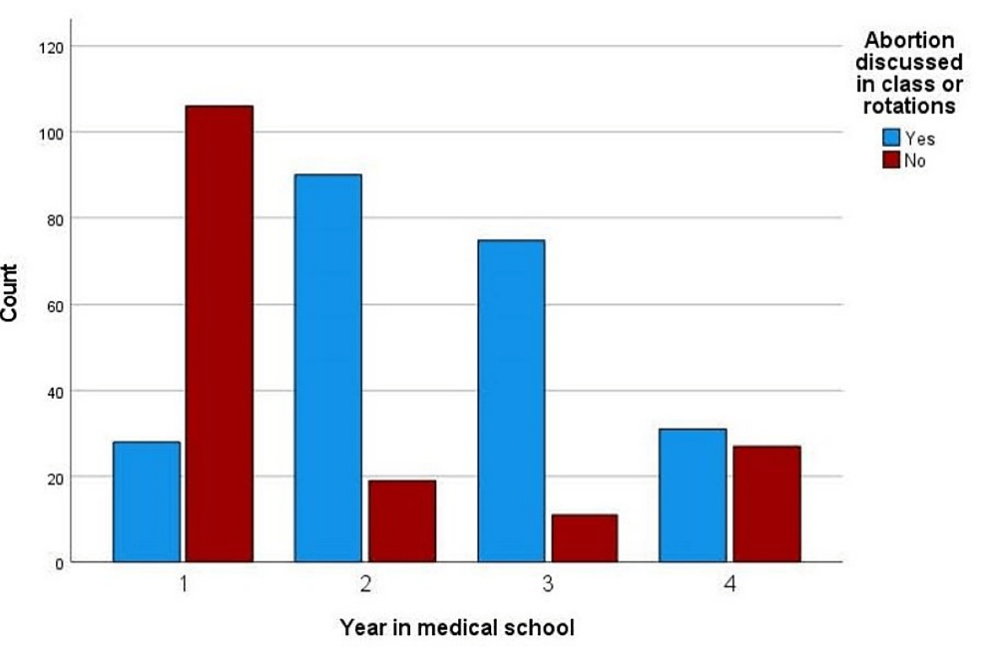
Abortion was a topic of discussion during medical school by year of training.

Figure 1 reports abortion being discussed during medical school by year of training. Of the participants who responded that abortion has been a topic of discussion in their school training (n = 387), 57.4% (n = 224) reported they had, with 20.9% (n = 28) of first-year students, 82.6% (n = 90) of second-year students, 82.7% (n = 75) of third-year students, and 53.4% (n = 31) of fourth-year students reporting abortion being discussed while in school.

**Figure 2:**
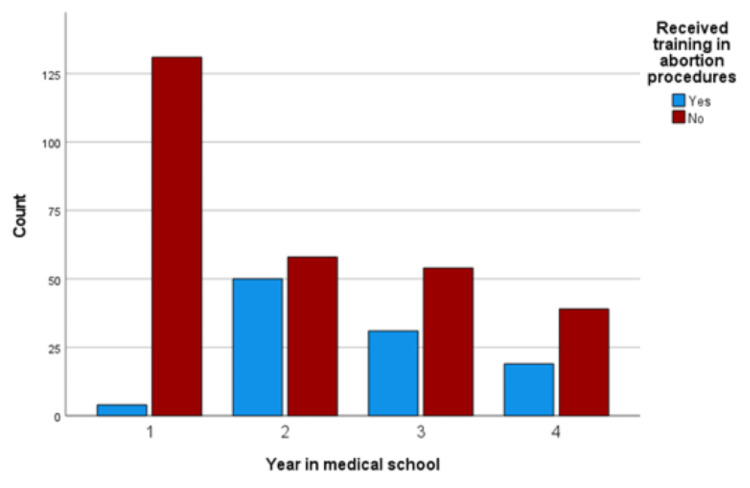
Students who received training in abortion practices, indications, or procedures.

Figure 2 reports students who received training in abortion practices, indications, or procedures. Of the participants who responded that they had received some training in abortion practices, indications, or procedures (n = 390), 26.7% (n = 104) reported they had, with 3% (n = 4) of first-year students, 46.3% (n = 50) of second-year students, 36.5% (n = 31) of third-year students, and 32.8% (n = 19) of fourth-year students reporting receiving any training in abortion practices, indications, or procedures while in school.

Table 2 reports the summary statistics for the major study variables. The mean score for the scale that measured attitudes about abortion was 4.67 (on a scale of 1 to 6), with higher scores indicating more positive attitudes toward abortion. The mean score for the religiosity scale was 3.0 (on a scale of 1 to 5), with higher scores indicating higher levels of religiosity. The mean score for the abortion knowledge scale was 7.43, with higher scores indicating greater levels of abortion knowledge. The mean score for the single item, “How important is religion in your life?,” was 2.44 (on a scale of 1 to 4), with higher scores indicating less importance of religion in their life.

**Table 2:** Summary statistics for the major study variables.

	n	Mean	Standard deviation	Minimum	Maximum
Attitudes about abortion	413	4.67	1.202	1	6
Religiosity	407	3.00	1.148	1	5
Abortion knowledge	397	7.43	2.010	1	9
Importance of religion in life	388	2.44	1.115	1	4

Table 3 reports the correlation analysis. The study variables that were hypothesized to be correlated with abortion attitudes were included in the analysis. Only the variables with significant correlations were added to the regression analysis.

**Table 3:** Correlation table. **: Correlation is significant at the 0.01 level (two-tailed). *: Correlation is significant at the 0.05 level (two-tailed).

	1	2	3	4	5	6
1. Attitudes about abortion	1.000					
2. Religiosity	0.454^**^	1.000				
3. Abortion knowledge	0.508^**^	0.206^**^	1.000			
4. Sex	0.299^**^	0.088	0.206^**^	1.000		
5. Importance of religion in life	0.436^**^	0.879^**^	0.182^**^	0.053	1.000	
6. Pro-choice/pro-life group identification	0.452^**^	0.398^**^	0.240^**^	-0.061	0.413^**^	1.000

Linear regression modeling indicated that religiosity, abortion knowledge, being a woman, and group identification regarding abortion (“pro-choice” or “pro-life”) explain a significant amount of the variance in abortion attitude scores in medical students (Table 4). Multicollinearity tests were performed and variance inflation factors were within range. A significant regression equation was found, F(6,373) = 83.570, p < 0.000, R^2^ = 0.603, R^2^ adjusted = 0.611). The percentage of variance in the scores accounted for by the model was 60%. The combination of independent variables (i.e., knowledge, religiosity, sex, pro-choice/pro-life group identification, and importance of religion) predicting even a moderate amount of variance in a relevant outcome could be significant. Less religiosity, greater abortion knowledge, being a woman, and identifying as “pro-choice” significantly contributed to more positive attitudes toward abortion in this sample of medical students. Interestingly, while moderately correlated with abortion attitudes (r = 0.436, p < 0.01), the single item regarding the importance of religion in one’s life did not contribute to the model.

**Table 4:** Regression model for predicting abortion attitudes in medical students. Predicted variable: abortion attitudes *p < 0.05, **p < 0.01.

	Unstandardized B	Standard error	Beta	t-statistic	Significance (p)
(Constant)	19.635	4.824		4.070	0.000**
Religiosity	1.099	0.280	0.268	3.925	0.000**
Abortion knowledge	3.677	0.425	0.298	8.646	0.000**
Sex (Male/Female)	8.002	1.660	0.161	4.821	0.000**
Pro-choice/pro-life group identification	21.596	1.961	0.395	11.011	0.000**
Importance of religion in life	1.652	1.431	0.078	1.155	0.249

## Discussion

The findings from this study suggested that abortion knowledge, level of religiosity, sex, and philosophical group affiliation (“pro-choice” vs. “pro-life”) significantly contributed to attitudes toward abortion in this sample of medical students in years one through four of undergraduate training. The level of self-reported importance of religion in one’s life did not contribute to the model. More than half of the participants reported that abortion had been discussed in medical school, but only a little more than one-fourth reported having received training in abortion practices, indications, or procedures.

Abortion training in medical school

A little more than half of the participants (n = 224; 57.4%) reported that abortion had been a topic of discussion in school training, and only about 27% (n = 104) received training in abortion practices, indications, or procedures. These findings are supported by previous studies that indicated that medical students have little formal abortion education, abortion-specific lectures, and discussions or clinical abortion care experience prior to clinical rotations [3]. While the participants in this current study were enrolled in medical school before the *Roe v. Wade* reversal, those numbers are less than optimal. It could be of value to review the next year or so to see if some if not all of the classroom discussions and clinical training have diminished since the new law. This could leave some medical students with sub-par education and training in abortion and could impact the quality of care provided by these future physicians should they practice in a U.S. state with less stringent restrictions on abortion.

“Pro-choice” vs. “pro-life” group affiliation

Regarding philosophical group affiliation when referring to abortion, a Gallup Poll of 1,007 adults conducted in May 2022 indicated that 55% self-identified as “pro-choice” and 34% self-identified as “pro-life” (5% had no opinion) [33]. The younger the respondent, the higher the percentage in the “pro-choice” group, with 71% of the respondents aged 18-29 years self-identifying as “pro-choice” [33]. In this study, 79.2% (n = 309) reported “pro-choice” and 16.9% (n = 66) reported “pro-life,” which is in alignment with the national statistics for this age group. The result that having a “pro-choice” affiliation predicts more positive attitudes toward abortion is not surprising and is supported by the aforenoted reports, with 79.2% (n = 309) of this young sample (mean age = 25.8 years) of medical students self-identifying as “pro-choice” [34].

Sex

Regarding sex, women represented 65.9% (n = 251) of the sample; published reports indicate that women born between the mid-1990s and mid-2010s are more likely to oppose restrictions on abortion than men. Interestingly, there were no differences between men and women in this sample regarding pro-choice/pro-life group affiliation. However, being a woman (and being “pro-choice” regardless of sex) contributed to the regression model predicting more positive attitudes about abortion. It would be expected that women have more positive attitudes toward abortion than men (legalized abortion ensures women the right to control their bodies), yet there is research to support that sex has no bearing on abortion attitudes. On the other hand, there is research to support that women show stronger approval for women’s autonomy in abortion decision-making, including fathers not having the right to prevent the mother from having an abortion, which was the case in the current study [35].

Religiosity

Current religious affiliation in the sample was quite varied. Among religious groups, opposition to the right of women to legal abortion has been largely associated with white Evangelical Protestants and other smaller conservative Christian groups [34]. Regarding the importance of religion in one’s life (which did not contribute to the model predicting abortion attitudes), about 42% (n = 201) reported religion was very or somewhat important; the rest reported that it was not too important or not important at all. It appears that religious affiliation or the importance of religion in one’s life may have little or no bearing on medical students’ attitudes toward abortion, which is not supported by previous research [36]. However, the level of religiosity (i.e., strong religious feeling or belief) predicted abortion attitudes in this sample. More research is needed to investigate the role of religious attitudes and how they may temper abortion attitudes among medical students and healthcare providers in general.

Abortion knowledge

It was thought that factual knowledge about abortion would be associated with more positive attitudes toward abortion. Linear regression modeling indicated that less religiosity, more knowledge about abortion, being a woman, and “pro-choice” affiliation explained 60% of the variance in the model predicting attitudes about abortion in this sample of medical students. The result that knowledge about abortion (i.e., scoring high on the knowledge questions) in conjunction with other factors such as being a woman, identifying as “pro-choice,” and low levels of religiosity (regardless of current religious affiliation) predicted positive attitudes toward abortion is noteworthy.

Implications for medical education and practice

While medical school educators strive to teach students *best practices* in all subject areas, only 22% of OB/GYN program graduates are competent in performing dilation and evacuation (the procedure for most second-trimester abortions) and only 71% of graduates are competent in performing first-trimester aspiration [37]. These percentages are disturbingly low, considering that one in four U.S. women will undergo an abortion [38]. In this sample, the mean score on the abortion knowledge index was 7.43 (on a scale of 0-10), with higher scores indicating more factual knowledge about abortion. Considering the medical students in this study were trained prior to the *Roe v. Wade* reversal (preclinically and clinically), this number is low.

Medical trainees have the capacity (or have been trained) to separate science from personal feelings for the good of the patient’s needs and preferences. Educators should strive to impart knowledge and provide thorough training in abortion practices and procedures (e.g., dilation and evacuation, medical abortion, medical management of miscarriage); however, with the recent reversal of *Roe v. Wade*, abortion training may be stained if not completely compromised. Moreover, the number of institutions where future Ob/GYNs can acquire this training will become limited [39]. It is, therefore, imperative that the standard of education not slide with the advent of political rulings that will be subject to differences in allowable practices on a state-to-state basis.

While studies show students believe their abortion education to be insufficient, students find their minimal experiences valuable, believe it will assist them in their future careers, and would recommend it to future students [20]. Additionally, researchers found that abortion vignettes as part of the medical school curriculum helped students grow professionally by identifying their own core values, recognize when those values conflict with their patients, provide a safe space to learn how to meet professional obligations/career responsibilities of humanism, respect patient privacy and autonomy, and resolve conflict [20].

Medical student knowledge and exposure to abortion in their medical curriculum may continue to decrease. With increasing restrictions in particular states in the U.S., students and residents may have even less exposure to abortion. In 2014 the American College of Obstetrics and Gynecology determined that more abortion education was needed in medical school and residency; however, legal and funding requirements made it difficult to train students [15]. After the *Dobbs v. Jackson Health* ruling, it has become even more difficult to train students as more facilities continue to close, nearly 44% of the 6,000 OB/GYN residents in the U.S. will not have access to in-state abortion training, and students will have even fewer despite the growing need in those states [21]. The lack of opportunities has caused some medical students and residents to seek overseas locations to obtain in-person abortion training [21], indicating the desire among trainees to have access to abortion training.

New legislation regarding abortion may have a profound impact on not only the attitudes of medical trainees but the nature in which they are trained when it comes to abortion practices, indications, or procedures. Some clinics where medical schools provide first-hand abortion experience have closed. As a surge of extreme restrictions on abortion has been seen in certain states in the U.S., medical schools and residency programs need to address these issues to ensure future physicians are adequately prepared.

Limitations

First, this study utilized a cross-sectional survey design to collect data, and thus generalizations cannot be made regarding changes or trends over time, the directionality of influence, or cause-and-effect relationships. Second, data were collected from one institution; multi-site data collection might have provided a more diverse sample of respondents. Third, there were several disadvantages of conducting research using online questionnaires, such as limited respondent availability or willingness to respond without the researcher present. Fourth, during the year this study took place, 15 states passed new laws regulating access to abortion, with Florida being one of the nine states that passed a 15-week ban on abortion [40]. The changes that came about from the Supreme Court’s decision that reversed *Roe v. Wade*, eliminating the constitutional right to an abortion, may have also influenced participants’ responses. Due to these issues, the generalizability of the findings cannot be made regarding all medical students across the U.S.

## Conclusions

The present study is the first, to our knowledge, to identify medical student characteristics (sex, philosophical group affiliation regarding abortion, level of factual knowledge about abortion, and religiosity) as indicators of abortion attitudes. With the reversal of *Roe v. Wade*, attention must be given to the possible change in medical students’ attitudes toward abortion (as well as any newly developed constraints on clinical training) and to ensure the provision of comprehensive education as U.S. states will determine the limits of these practices and procedures. While further research in this area is needed, findings from this study can help assess students’ attitudes about abortion and guide medical education efforts to better prepare tomorrow’s OB/GYN physicians.
